# Serum Anti-Aminoacyl-Transfer Ribonucleic Acid Synthetase Antibody Levels Are Involved in Rheumatoid Arthritis Complicated with Interstitial Lung Disease

**DOI:** 10.3390/jcm13226761

**Published:** 2024-11-10

**Authors:** Shomi Oka, Takashi Higuchi, Hiroshi Furukawa, Kota Shimada, Akira Okamoto, Misuzu Fujimori, Atsushi Hashimoto, Akiko Komiya, Koichiro Saisho, Norie Yoshikawa, Masao Katayama, Toshihiro Matsui, Naoshi Fukui, Kiyoshi Migita, Shigeto Tohma

**Affiliations:** 1 Department of Rheumatology, NHO Tokyo National Hospital, Kiyose 204-8585, Japan; oka-tkb@umin.org (S.O.); takashi.qef@ac.auone-net.jp (T.H.); touma.shigeto.jy@mail.hosp.go.jp (S.T.); 2 Clinical Research Center for Allergy and Rheumatology, NHO Sagamihara National Hospital, Sagamihara 252-0392, Japan; komiya.akiko.zy@mail.hosp.go.jp (A.K.); ninja02matsui@gmail.com (T.M.); n-fukui@idaten.c.u-tokyo.ac.jp (N.F.); 3 Department of Rheumatology, NHO Sagamihara National Hospital, Sagamihara 252-0392, Japan; kouta_shimada@tmhp.jp (K.S.); hako@happy.email.ne.jp (A.H.); 4 Department of Rheumatic Diseases, Tokyo Metropolitan Tama Medical Center, Fuchu 183-8524, Japan; 5 Department of Rheumatology, NHO Himeji Medical Center, Himeji 670-8520, Japanmisuzufujimori@yahoo.co.jp (M.F.); 6 Department of Internal Medicine, Sagami Seikyou Hospital, Sagamihara 252-0303, Japan; 7 Department of Clinical Laboratory, NHO Sagamihara National Hospital, Sagamihara 252-0392, Japan; 8 Department of Orthopedics/Rheumatology, NHO Miyakonojo Medical Center, Miyakonojo 885-0014, Japan; saisho889@miyazaki-catv.ne.jp (K.S.); nori1004@me.com (N.Y.); 9 Tanimura Hospital, Nobeoka 882-0041, Japan; 10 Department of Internal Medicine, NHO Nagoya Medical Center, Nagoya 460-0001, Japan; 11 Department of Life Sciences, Graduate School of Arts and Sciences, The University of Tokyo, Tokyo 153-890, Japan; 12 Clinical Research Center, NHO Nagasaki Medical Center, Omura 856-8562, Japan; k-migita@sfh.or.jp; 13 Department of Gastroenterology and Rheumatology, Fukushima Medical University School of Medicine, Fukushima 960-1295, Japan

**Keywords:** rheumatoid arthritis, anti-aminoacyl-transfer ribonucleic acid synthetase antibodies, interstitial lung disease

## Abstract

**Objectives:** A common complication in patients with rheumatoid arthritis (RA) is interstitial lung disease (ILD). Antibodies (Abs) to anti-aminoacyl-transfer ribonucleic acid synthetase (ARS) are linked to ILD in patients with idiopathic inflammatory myopathies (IIM). There have been limited studies of anti-ARS Abs in RA. In this study, we examined anti-ARS Abs in ILD in patients with RA. **Methods:** Anti-ARS Abs in serum from patients with RA were measured. **Results:** There were higher anti-ARS Ab levels in RA patients with ILD (mean ± SDM, 16.3 ± 32.3 vs. 7.4 ± 7.0 (Index), *p* = 5.58 × 10^−12^), usual interstitial pneumonia (14.4 ± 24.4 vs. 7.4 ± 7.0 [Index], *p* = 3.14 × 10^−12^), and nonspecific interstitial pneumonia (17.9 ± 37.7 vs. 7.4 ± 7.0 (Index), *p* = 5.07 × 10^−5^) compared with patients without chronic lung disease. The area under the curve (AUC) of the receiver operating characteristic curve for anti-ARS Ab was too low to allow for discrimination among RA patients with/without chronic lung disease (0.608, 95% confidence interval (CI) 0.560–0.655, *p* = 8.69 × 10^−6^). Multiple logistic regression analyses of age, smoking status, anti-ARS Abs, as well as Steinbrocker stage generated an ARS-index with a high AUC value (0.707, 95%CI 0.662–0.752, *p* = 2.20 × 10^−19^). **Conclusions:** Anti-ARS Abs are related to ILD pathogenesis in RA and may be a biomarker for ILD.

## 1. Introduction

Rheumatoid arthritis (RA), characterized by synovial joint destruction, is a representative systemic inflammatory disease [1]. It can be complicated with extra-articular manifestations such as chronic lung diseases (CLD), including interstitial lung disease (ILD), emphysema, and airway disease (AD) [2]. Unfortunately, patients with RA complicated with ILD or AD often have a prognosis that is poor [3,4,5,6,7]. The usual interstitial pneumonia (UIP) pattern of ILD confers a poor prognosis in patients with RA [8]. Thus, the pathogenesis of ILD and AD in RA should be elucidated. The term acute-onset diffuse ILD (AoDILD) covers a broad range of disorders that frequently occur in RA patients, including drug-induced ILD, acute exacerbation of ILD, and pneumocystis pneumonia, which confer a poor prognosis [9,10].

Antibodies (Abs) that recognize the Fc of immunoglobulin G are designated as rheumatoid factors (RFs), and Abs against citrullinated peptides are termed anti-citrullinated peptide antibodies (ACPAs). ACPA and RF are specific for RA and, therefore, can aid the diagnosis of RA [11]. RF and ACPA were reported to be involved in ILD in RA [12,13,14,15] and are considered auto-Ab biomarkers of ILD in RA [16]. The production of Abs to anti-melanoma differentiation-associated gene 5, which recognizes RNA helicase, was linked to clinically amyopathic dermatomyositis with rapidly progressive ILD [17]. However, it was reported that these auto-Abs were present in RA patients with AD [18]. Auto-Abs against enzymes that attach specific amino acids to transfer ribonucleic acid (tRNA) present in idiopathic inflammatory myopathies (IIM) are designated anti-aminoacyl-tRNA synthetase (ARS) Abs. Anti-ARS Abs include antibodies against eight antigens (Jo1 [histidyl tRNA synthetases], PL-7 [threonyl tRNA synthetases], PL-12 [alanyl tRNA synthetases], EJ [glycyl tRNA synthetases], OJ [isoleucyl tRNA synthetases], KS [asparaginyl tRNA synthetases], Zo [phenylalanyl tRNA synthetases], and Ha [tyrosyl tRNA synthetases]) and were specifically detected as myositis-specific Abs in patients with IIM [19,20]. ILD is frequently complicated in patients with IIM who also have anti-ARS Abs [20]. Anti-synthetase syndrome, a clinical subset of IIM, is categorized by mechanic’s hands, ILD, myositis, arthritis, the production of anti-ARS Abs, fever, and Raynaud’s phenomenon [21]. ILD in IIM patients with anti-ARS Abs was reported to progress slowly but did not confer a poor prognosis in patients [22]. Anti-ARS Abs were also detected in idiopathic interstitial pneumonia [23,24,25,26,27], but not RA [19,27,28]. Another study analyzed anti-ARS Abs qualitatively and showed they were involved in ILD with RA [29]. Limited studies have examined anti-ARS Abs in RA or other autoimmune diseases. Here, anti-ARS Abs were quantitated and their association with ILD was investigated in patients with RA.

## 2. Materials and Methods

### 2.1. Patients

Five hundred and fifty-eight RA patients with chest computed tomography findings and fifty-two healthy controls were recruited at Tokyo Hospital, Sagamihara Hospital, Nagoya Medical Center, Miyakonojo Medical Center, Nagasaki Medical Center, and Himeji Medical Center. All patients with RA fulfilled the American College of Rheumatology criteria for RA [30] or rheumatoid arthritis classification criteria [11]. Predominant chest computed tomography findings of RA patients [31] were used to diagnose emphysema, nonspecific interstitial pneumonia (NSIP), AD, no CLD, or UIP (Supplementary Figure S1). The ILD groups included UIP and NSIP patients and the CLD (+) group included NSIP, emphysema, AD, and UIP. The production of anti-ARS Abs was analyzed using serum collected from patients with RA and controls. The 558 RA patients included 1 case complicated with polymyositis, 1 with dermatomyositis, 1 with scleroderma, and 1 with systemic lupus erythematosus.

Eleven RA patients with AoDILD were treated with corticosteroid pulse therapy at Sagamihara Hospital (mean age at admission ± standard deviation [SD], 65.9 ± 8.1 years, five male patients). These patients fulfilled the American College of Rheumatology RA criteria 30. Five had an acute exacerbation of ILD and six had drug-induced ILD; the definitions of these cases were previously described [10]. Sera from patients with RA with AoDILD were obtained at admission and under stable conditions at least three months before admission.

This study was reviewed and approved by the Research Ethics Committees of Tokyo Hospital (190010, 29 May 2019), Sagamihara Hospital, and the Central Institutional Review Board of the National Hospital Organization. Written informed consent was obtained from all study participants except for those who were deceased before the initiation of the study. Any serum samples obtained before this study were anonymized to prevent identifying participants and analyses were only approved by the Sagamihara National Hospital Research Ethics Committee based on this condition. This study was conducted in accordance with the principles of the Declaration of Helsinki.

### 2.2. Detection of Anti-ARS Abs

Serum anti-ARS Abs were measured by enzyme-linked immunosorbent assay (ELISA, Mesacup anti-ARS tests, Medical & Biological Laboratories, Tokyo, Japan) according to the manufacturer’s instructions. Index values = (sample absorbance—negative control absorbance)/(positive control absorbance—negative control absorbance) × 100. The cut-off value was 11.374 based on the 98th percentile of healthy controls (*n* = 52), although the cut-off level recommended by the kit manufacturer was 25. To discriminate between anti-Jo1 Abs, anti-PL7 Abs, anti-PL12 Abs, anti-EJ Abs, and anti-OJ Abs, anti-ARS Abs were also detected by a line blot assay (Euroline Myositis Profile 3, Euroimmun AG, Lübeck, Germany) using patient sera positive for anti-ARS Abs as determined by ELISA. Clinical information of some RA patients was previously described [18]. Steinbrocker stages were assessed as previously reported [32].

### 2.3. Statistical Analysis

Anti-ARS Abs titers between RA patients without CLD or controls were compared by the Mann–Whitney U-test. Fisher’s exact test with 2 × 2 contingency tables was used to compare the presence of anti-ARS Abs in RA patients without CLD. Multiple logistic regression analyses were used to generate an ARS-index from anti-ARS Ab, smoking status [never smoker: 0; past smoker: 1; current smoker: 2], Steinbrocker stage [1,2,3,4], and age [years]. To compare RA patients with/without CLD, receiver operating characteristic (ROC) curves for anti-ARS Abs or the ARS-index were used. The area under the curve (AUC) values of ROC curves were compared by the chi-squared test. Cut-off levels were optimized on the basis of the highest Youden’s index. Anti-ARS Ab levels in serum from RA patients with stable lung disease and AoDILD conditions were tested by the Wilcoxon signed-rank test.

## 3. Results

### 3.1. Comparisons of Anti-ARS Abs in RA Subsets with CLD (−) RA Patients

Anti-ARS Abs in RA patient sera detected by ELISA and Ab levels in subsets underwent a comparison with those in the CLD (−) group (Supplementary Figure S2 and Table 1). Anti-ARS Ab levels were increased in RA with ILD (mean ± SD, 16.3 ± 32.3 vs. 7.4 ± 7.0 [Index], *p* = 5.58 × 10^−12^), UIP (14.4 ± 24.4 vs. 7.4 ± 7.0 [Index], *p* = 3.14 × 10^−12^), and NSIP (17.9 ± 37.7 vs. 7.4 ± 7.0 [Index], *p* = 5.07 × 10^−5^). Anti-ARS Ab levels were also increased in RA and CLD (11.2 ± 21.4 vs. 7.4 ± 7.0 [Index], *p* = 1.77 × 10^−5^). This indicated that anti-ARS Ab titers were linked with ILD, UIP, NSIP, and CLD in RA.

Evaluations of RA patients that might have been positive for anti-ARS Abs used a cut-off value based on the results from healthy controls (Table 2). Anti-ARS Ab positivity was associated with ILD (*n* [%], 47 [34.1%] vs. 18 [8.4%], *p* = 1.08 × 10^−13^), UIP (25 [39.7%] vs. 18 [8.4%], *p* = 1.15 × 10^−11^), NSIP (22 [29.3%] vs. 18 [8.4%], *p* = 2.63 × 10^−8^), and emphysema (9 [23.1%] vs. 18 [8.4%], *p* = 0.0003) in RA. Anti-ARS Ab positivity was also increased in RA with CLD (70 [20.4%] vs. 18 [8.4%], *p* = 1.73 × 10^−8^). Evaluations of anti-ARS Abs positivity in RA patients used the recommended cut-off value (Supplementary Table S2). Anti-ARS Ab positivity was associated with ILD (*n* [%], 10 [7.2%] vs. 3 [1.4%], *p* = 0.0070) and NSIP (7 [9.3%] vs. 3 [1.4%], *p* = 0.0037) in RA. The precise profiles of anti-ARS Abs were analyzed by line blot assay in 16 RA patient sera positive for anti-ARS Abs by ELISA (Supplementary Table S2); these RA patients included 1 with polymyositis, 1 with dermatomyositis, and 1 with scleroderma. Anti-ARS Abs were detected in 12 patients (75.0%) by line blot assays of samples from 16 patients. The sera from two RA patients with UIP and one with NSIP were positive for two or more anti-ARS Abs by line blot assay. Anti-PL7 Abs were found in all RA patients with UIP.

### 3.2. Comparison of Anti-ARS Abs Between RA Patients and Healthy Controls

A comparison of anti-ARS Ab levels demonstrated increased levels in all RA subsets and RA, per se, compared with healthy controls (*n* = 52, 5.3 ± 2.1 [Index], Supplementary Figure S2 and Table 3).

### 3.3. Multiple Logistic Regression Analysis of Anti-ARS Ab

Multiple logistic regression analyses were performed to eliminate the effects of clinical manifestation on the association of anti-ARS Abs with CLD (Supplementary Table S3). The association of anti-ARS Abs with CLD was observed in univariate analysis (*p* = 0.0241, odds ratio [OR] 1.04, 95% confidence interval [CI] 1.01–1.08), and it remained significant (P_adjusted_ = 0.0204, OR_adjusted_ 1.04, 95% CI 1.01–1.08),when conditioned on age, Steinbrocker stage, and smoking status. Thus, these data suggested the independent association of anti-ARS Abs with CLD in RA.

Multiple logistic regression analyses were also conducted to eliminate the possibility of confounding the association of anti-ARS Abs between RA and healthy controls (Supplementary Table S4). The association was revealed to be independent of age and sex.

### 3.4. ROC Analyses

**Figure 1:**
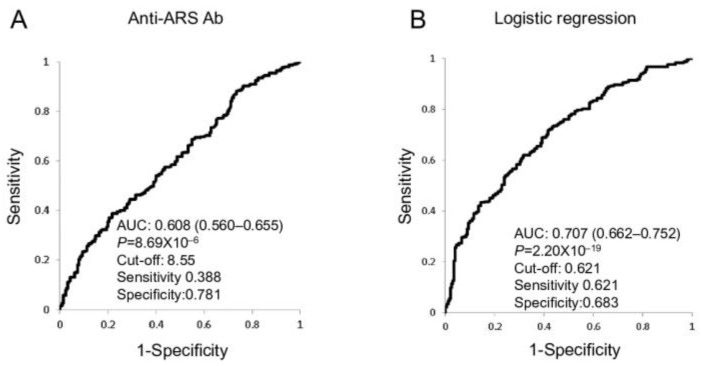
ROC curves using anti-ARS Abs and multiple logistic regression analysis to compare CLD (+) and CLD (−) RA. ROC curves for anti-ARS Abs (**A**) and multiple logistic regression analysis (**B**) with anti-ARS Abs (Index), Steinbrocker stage (1–4), age (years), and smoking status (never smoker: 0; past smoker: 1; current smoker: 2) were calculated. The AUC values of the ROC curves with 95% CIs and the cut-off levels are shown. RA: rheumatoid arthritis; ARS: aminoacyl-transfer ribonucleic acid synthetase; CLD: chronic lung disease; Ab: antibody; ROC: receiver operating characteristic; AUC: area under the curve; CI: confidence interval.

A ROC curve of anti-ARS Ab was used for comparisons between RA with/without CLD (Figure 1A). The AUC of the ROC curve for anti-ARS Ab (0.608, 95% confidence interval [CI] 0.560–0.655, *p* = 8.69 × 10^−6^) was not high enough for clinical use as a biomarker. Multiple logistic regression analysis of age, anti-ARS Abs, smoking status, and Steinbrocker stage was performed to generate an ARS-index: 0.0415 × (anti-ARS Abs) + 0.0565 × (age)—0.1917 × (Steinbrocker stage) + 0.4560 × (smoking status) − 3.3217. The AUC of the ROC curve was 0.707 (95% CI 0.662–0.752, *p* = 2.20 × 10^−19^, Figure 1B) and was significantly greater than that for anti-ARS Abs (*p* = 0.0024). An ARS-index with a greater AUC value was generated by multiple logistic regression analysis of age, anti-ARS Abs, smoking status, and Steinbrocker stage.

### 3.5. Detection of Anti-ARS Abs in RA Patients with AoDILD

**Figure 2:**
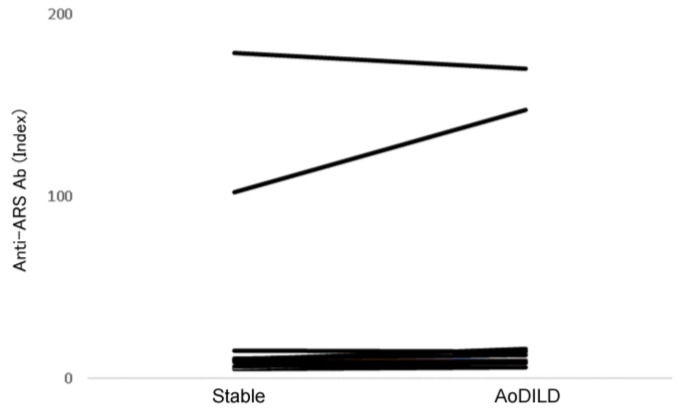
Anti-ARS Ab levels in sera from RA patients with AoDILD. Anti-ARS Ab levels in sera from RA patients under stable and AoDILD conditions are shown. Differences were tested using the Wilcoxon signed-rank test. AoDILD: acute-onset diffuse interstitial lung disease; RA: rheumatoid arthritis; ARS: aminoacyl-transfer ribonucleic acid synthetase; Ab: antibody.

Anti-ARS Abs measured in sera from patients with RA and AoDILD indicated that two RA patients were positive (Figure 2). No difference was found between RA patients under stable or AoDILD conditions. Anti-ARS Abs in two RA patients with AoDILD were analyzed by line blot assay; one patient had anti-PL7 Abs, anti-PL12 Abs, and anti-Jo1Abs, and the other had anti-Jo1Abs. The Ab profiles of the two patients under stable conditions were the same as those in patients with RA and AoDILD.

## 4. Discussion

The current study showed that anti-ARS Abs were involved in ILD, NSIP, and UIP in patients with RA. The AUC value of ROC curves for anti-ARS Abs was not sufficiently high for clinical use as a biomarker for the comparison between RA with/without CLD. An ARS-index was generated using age, anti-ARS Abs, smoking status, and Steinbrocker stage, and the AUC value of the ROC curve for the ARS-index was higher than that for anti-ARS Ab alone.

A correlation between several auto-Abs and ILD was previously reported for RA [12,13,14,15]. We noted that anti-ARS Abs were associated with ILD in RA. A relationship between anti-ARS Abs and UIP and NSIP was demonstrated in this study, suggesting the participation of anti-ARS Abs in the common pathogenesis of UIP and NSIP in RA. The functions of individual ACPA, RF, and anti-ARS Abs in the pathogenesis of ILD in RA might be revealed in the future.

Anti-ARS Abs were reported in idiopathic interstitial pneumonia patients without myopathy [23,24,25,26,27], but not in RA patients [19,27,28]. Anti-ARS Abs were reportedly present in 6.1% of RA patients by a line blot assay and these were related to ILD RA [29]. We observed that 15.8% of RA patients developed anti-ARS Abs as measured by ELISA using our cut-off level, which confirmed an association between anti-ARS Abs and ILD. Our findings suggest that ELISA has higher sensitivities than the line blot assay.

Multiple logistic regression analysis showed the independent association of anti-ARS Abs from age, Steinbrocker stage, and smoking status. However, it is still possible that other clinical factors would influence the associations.

An ARS index was generated by multiple logistic regression analysis using age, smoking status, anti-ARS Abs, and Steinbrocker stage. The AUC value of the ROC curve of the ARS-index was greater than that for anti-ARS Abs, suggesting anti-ARS Abs might be useful complex biomarkers for CLD in patients with RA. Since ILD is heterogeneous, other antibodies could be involved in the pathogenesis of other subsets of ILD in RA. Thus, other antibodies should be investigated to improve the index for clinical use.

The cut-off level in this study was set for anti-ARS Ab positivity (11.374) and it was lower than the recommended cut-off level (25). However, our cut-off level was better for the discrimination of ILD in RA patients. Anti-ARS Ab levels ≥ 25 were observed in 16 RA patients in the ELISA; each anti-ARS Ab was detected in 12 of these RA patients in the line blot assay. Anti-PL-7 Abs were detected in all RA patients with UIP. The 16 RA patients included 1 with polymyositis, 1 with dermatomyositis, and 1 with scleroderma. The distribution patterns of anti-ARS Abs in RA differed from those in IIM [33], suggesting different pathogenesis of ILD between patients with RA and IIM. Thus, anti-ARS Abs might be useful biomarkers for ILD or CLD in RA.

Anti-ARS Abs were present in RA with AoDILD although ILD in IIM patients with anti-ARS Abs progressed slowly [22]. These results suggested different functions of anti-ARS Abs in RA and IIM.

Because a modest sample size was used, large-scale replicating studies of anti-ARS Abs in patients with RA should be conducted on multi-ethnic populations. Since anti-ARS Abs were analyzed in only patients with RA in the present study, anti-ARS Ab profiles also should be analyzed in other autoimmune disease patients. Because the information on the prognosis of RA patients with ILD was not available, we were not able to evaluate whether the disease progression of ILD in RA patients with anti-ARS Abs was slow or rapid.

## 5. Conclusions

This quantitative analysis of anti-ARS Abs in RA suggests an association between anti-ARS Abs and ILD.

## Figures and Tables

**Table 1:** Anti-ARS Ab levels in RA patients.

	*n*	Anti-ARS Ab, Index (SD)	*p*
ILD	138	16.3 (32.3)	5.58 × 10^−12^
UIP	63	14.4 (24.4)	3.14 × 10^−12^
NSIP	75	17.9 (37.7)	5.07 × 10^−5^
Airway disease	166	7.1 (3.5)	0.3559
Emphysema	39	10.4 (12.7)	0.2131
CLD (+)	343	11.2 (21.4)	1.77 × 10^−5^
CLD (−)	215	7.4 (7.0)	

RA: rheumatoid arthritis; ARS: aminoacyl-transfer ribonucleic acid synthetase; UIP: usual interstitial pneumonia; NSIP: nonspecific interstitial pneumonia; ILD: interstitial lung disease; AD: airway disease; CLD: chronic lung disease; Ab: antibody. ILD group includes UIP and NSIP groups. The CLD (+) group includes UIP, NSIP, AD, and emphysema patients. The average values for each group are shown. Standard deviations are shown in parentheses. Differences compared with the CLD (−) population were tested using the Mann–Whitney *U*-test.

**Table 2:** The positivity of anti-ARS Ab in RA patients.

	Anti-ARS Ab Positive, *n* (%)	*p*
ILD	47 (34.1)	1.08 × 10^−13^
UIP	25 (39.7)	1.15 × 10^−11^
NSIP	22 (29.3)	2.63 × 10^−8^
AD	14 (8.4)	0.1272
Emphysema	9 (23.1)	0.0003
CLD (+)	70 (20.4)	1.73 × 10^−8^
CLD (−)	18 (8.4)	

RA: rheumatoid arthritis; ARS: aminoacyl-transfer ribonucleic acid synthetase; ILD: interstitial lung disease; UIP: usual interstitial pneumonia; NSIP: nonspecific interstitial pneumonia; AD: airway disease; CLD: chronic lung disease; Ab: antibody. ILD group includes UIP and NSIP groups. The CLD (+) group includes UIP, NSIP, AD, and emphysema patients. The cut-off value was set to 11.374 based on the 98th percentile of 52 healthy controls. The value for each group is shown. Percentages are shown in parentheses. Differences compared with the CLD (−) population were tested using Fisher’s exact test using 2 × 2 contingency tables.

**Table 3:** The anti-ARS Ab levels in RA subsets or overall RA patients were compared with healthy controls.

	*p*
ILD	3.83 × 10^−12^
UIP	3.85 × 10^−13^
NSIP	2.34 × 10^−7^
AD	9.12 × 10^−6^
Emphysema	0.0002
CLD (+)	2.58 × 10^−9^
CLD (−)	0.0007
RA	2.61 × 10^−7^

RA: rheumatoid arthritis; ARS: aminoacyl-transfer ribonucleic acid synthetase; UIP: usual interstitial pneumonia; NSIP: nonspecific interstitial pneumonia; ILD: interstitial lung disease; AD: airway disease; CLD: chronic lung disease; Ab: antibody. Differences compared with the healthy control group were tested using the Mann–Whitney *U*-test.

## Data Availability

The data that support the findings of this study are available in the paper and the Supplementary File. Other data are available from the authors upon reasonable request. However, the clinical information and antibody data of each participant are not available under the condition of informed consent acquisition, which was mandated by the Act on the Protection of Personal Information.

## Supplementary Materials

The following supporting information can be downloaded at: https://www.mdpi.com/article/10.3390/jcm13226761/s1, Table S1: Clinical manifestations of patients with rheumatoid arthritis. Table S2: The positivity of anti-ARS Ab in RA patients determined by ELISA or line blot assay. Table S3: Multiple logistic regression analysis of anti-ARS Ab levels and clinical manifestations of CLD in RA. Table S4. Multiple logistic regression analysis of anti-ARS Ab levels between RA patients and healthy controls. Figure S1: Flow chart depicting rheumatoid arthritis patients examined with chest computed tomography. Figure S2: Evaluation of anti-ARS Abs in patients with RA and controls.
