# Supplementary material for: Serum Anti-Aminoacyl-Transfer Ribonucleic Acid Synthetase Antibody Levels Are Involved in Rheumatoid Arthritis Complicated with Interstitial Lung Disease
Source: J Clin Med. 2024 Nov 10;13(22):6761. doi: 10.3390/jcm13226761 (PMC11594691; doi:10.3390/jcm13226761)
Supplement: Supplementary file 1 [file jcm-13-06761-s001.zip › Anti-ARS Ab#12Figure-S1.pdf]

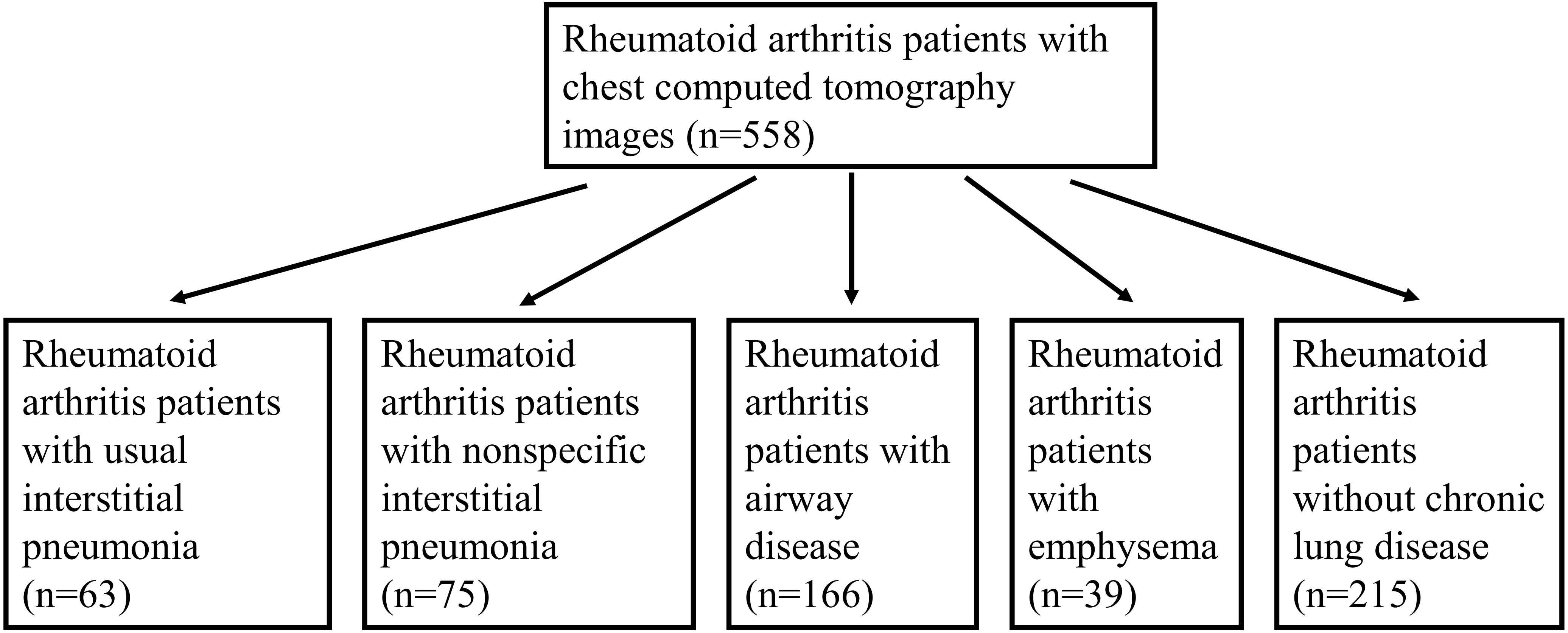

Supplementary Figure S1. Flow chart depicting rheumatoid arthritis patients examined with chest computed tomography.
