# Supplementary material for: Serum Anti-Aminoacyl-Transfer Ribonucleic Acid Synthetase Antibody Levels Are Involved in Rheumatoid Arthritis Complicated with Interstitial Lung Disease
Source: J Clin Med. 2024 Nov 10;13(22):6761. doi: 10.3390/jcm13226761 (PMC11594691; doi:10.3390/jcm13226761)
Supplement: Supplementary file 1 [file jcm-13-06761-s001.zip › Anti-ARS Ab#12Figure-S2.pdf]

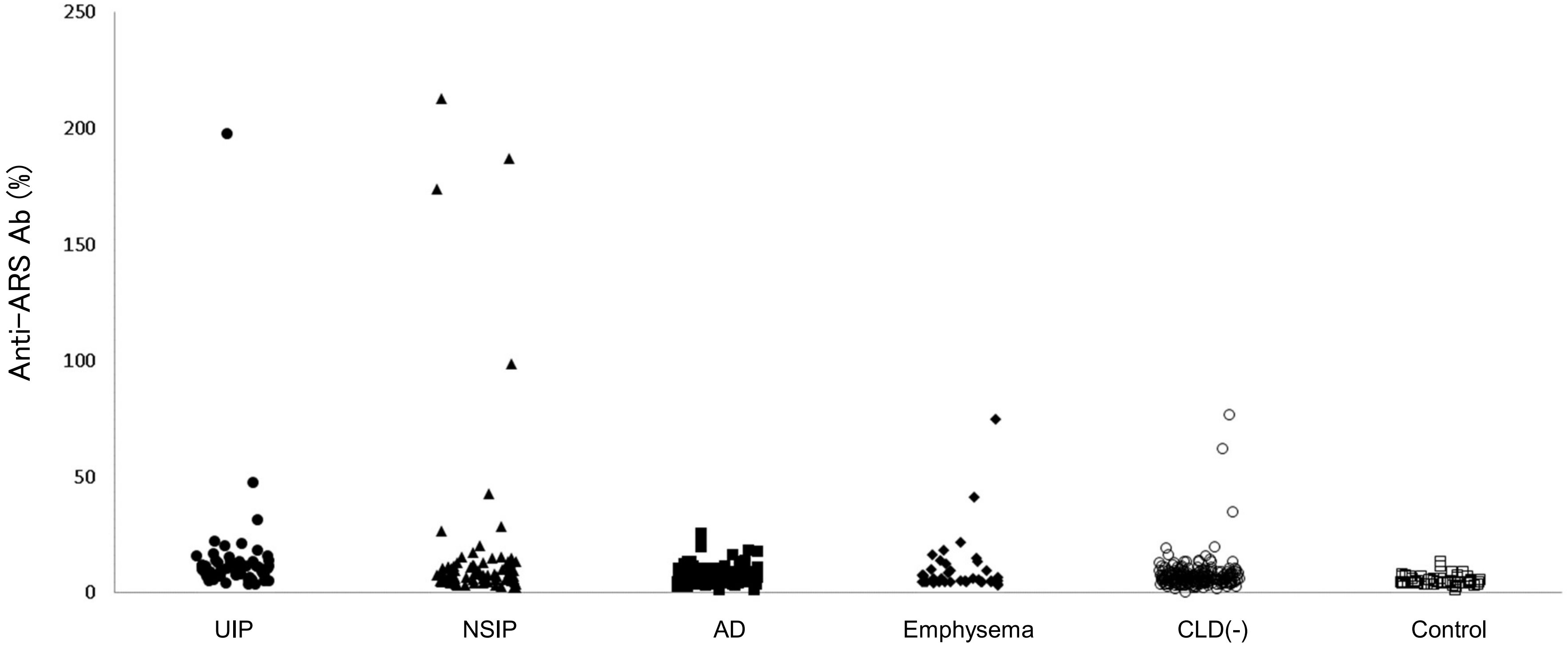

Supplementary Figure S2. Evaluation of anti-ARS Abs in patients with RA and controls. The distribution of anti-ARS Ab levels is shown. Filled circles, filled triangles, filled squares, filled diamonds, empty circles, and empty squares represent RA with UIP, RA with NSIP, RA with AD, RA with emphysema, RA without CLD, and controls, respectively. RA: rheumatoid arthritis, ARS: aminoacyl-transfer ribonucleic acid synthetase, UIP: usual interstitial pneumonia, NSIP: nonspecific interstitial pneumonia, AD: airway disease, CLD: chronic lung disease, Ab: antibody.
