# Supplementary material for: Serum Anti-Aminoacyl-Transfer Ribonucleic Acid Synthetase Antibody Levels Are Involved in Rheumatoid Arthritis Complicated with Interstitial Lung Disease
Source: J Clin Med. 2024 Nov 10;13(22):6761. doi: 10.3390/jcm13226761 (PMC11594691; doi:10.3390/jcm13226761)
Supplement: Supplementary file 1 [file jcm-13-06761-s001.zip › Anti-ARS Ab#12Table-S1.pdf]

Supplementary Table S1. Clinical manifestations of patients with rheumatoid arthritis.

	Usual interstitial pneumonia (n=63)	Nonspecific interstitial pneumonia (n=75)	Airway disease (n=166)	Emphysema (n=39)	Chronic lung disease(-) (n=215)
Mean age, years (SD)	70.0 (10.0)	67.5 (8.1)	67.9 (10.5)	66.8 (8.2)	62.4 (11.1)
Male, n (%)	23 (36.5)	14 (18.7)	28 (16.9)	24 (61.5)	36 (16.7)
Steinbrocker stage I, n (%)	21 (34.4)	26 (34.7)	21 (18.9)	14 (37.8)	31 (17.3)
Steinbrocker stage II, n (%)	11 (18.0)	20 (26.7)	25 (22.5)	10 (27.0)	47 (26.3)
Steinbrocker stage III, n (%)	8 (13.1)	8 (10.7)	12 (10.8)	6 (16.2)	30 (16.8)
Steinbrocker stage IV, n (%)	21 (34.4)	21 (28.0)	53 (47.7)	7 (18.9)	71 (39.7)
Never moker, n (%)	31 (52.5)	43 (60.6)	59 (59.0)	5 (15.2)	115 (69.3)
Past smoker, n (%)	24 (40.7)	17 (23.9)	13 (13.0)	19 (57.6)	31 (18.7)
Current smoker, n (%)	4 (6.8)	11 (15.5)	28 (28.0)	9 (27.3)	20 (12.0)
