# Supplementary material for: Serum Anti-Aminoacyl-Transfer Ribonucleic Acid Synthetase Antibody Levels Are Involved in Rheumatoid Arthritis Complicated with Interstitial Lung Disease
Source: J Clin Med. 2024 Nov 10;13(22):6761. doi: 10.3390/jcm13226761 (PMC11594691; doi:10.3390/jcm13226761)
Supplement: Supplementary file 1 [file jcm-13-06761-s001.zip › Anti-ARS Ab#12Table-S2.pdf]

Supplementary Table S2. The positivity of anti-ARS Ab in the RA patients with ELISA or line blot assay.

	Anti-ARS Ab positive by ELISA, n (%)	*P*	Anti-ARS Ab positive by line blot, n (%)	Anti-Jo1 Ab positive, n (%)	Anti-PL7 Ab positive, n (%)	Anti-PL12 Ab positive, n (%)	Anti-EJ Ab positive, n (%)	Anti-OJ Ab positive, n (%)
ILD	10 (7.2)	0.0070	9/10 (90.0)	3 (30.0)	4 (40.0)	3 (30.0)	2 (20.0)	1 (10.0)
UIP	3 (4.8)	0.1320	3/3 (100.0)	1 (33.3)	3 (100.0)	1 (33.3)	1 (33.3)	0 (0.0)
NSIP	7 (9.3)	0.0037	6/7 (85.7)	2 (28.6)	1 (14.3)	2 (28.6)	1 (14.3)	1 (14.3)
AD	1 (0.6)	0.6354	1/1 (100.0)	0 (0.0)	0 (0.0)	1 (100.0)	0 (0.0)	0 (0.0)
Emphysema	2 (5.1)	0.1699	1/2 (50.0)	0 (0.0)	0 (0.0)	0 (0.0)	0 (0.0)	1 (50.0)
CLD(+)	13 (3.8)	0.1213	11/13 (84.6)	3 (23.1)	4 (30.8)	4 (30.8)	2 (15.4)	2 (15.4)
CLD(—)	3 (1.4)		1/3 (33.3)	0 (0.0)	1 (33.3)	0 (0.0)	0 (0.0)	0 (0.0)

The positivity of anti-ARS Ab in RA patients determined by ELISA or line blot assay.

RA: rheumatoid arthritis, ILD: interstitial lung disease, UIP: usual interstitial pneumonia, NSIP: nonspecific interstitial pneumonia, AD: airway disease, CLD: chronic lung disease, ELISA: enzyme-linked immunosorbent assay. The ILD group includes UIP and NSIP patients. The CLD(+) group includes UIP, NSIP, AD, and emphysema patients. The cut-off value was set to 25 by the kit manufacturer.The number of each group is shown. Percentages are shown in parentheses. Differences compared with the CLD(—) population were tested using Fisher's exact test using 2 × 2 contingency tables.
