# Supplementary material for: Serum Anti-Aminoacyl-Transfer Ribonucleic Acid Synthetase Antibody Levels Are Involved in Rheumatoid Arthritis Complicated with Interstitial Lung Disease
Source: J Clin Med. 2024 Nov 10;13(22):6761. doi: 10.3390/jcm13226761 (PMC11594691; doi:10.3390/jcm13226761)
Supplement: Supplementary file 1 [file jcm-13-06761-s001.zip › Anti-ARS Ab#12Table-S3.pdf]

Supplementary Table S3. Multiple logistic regression analysis of anti-ARS Ab levels and clinical manifestations for CLD in RA.

Clinical manifestations	Unconditioned			Conditioned on the other factors		
	OR	95%CI	*P*	$OR_{adjusted}$	95%CI	$P_{adjusted}$
Anti-ARS Ab, Index	1.04	(1.01-1.08)	0.0241	1.04	(1.01-1.08)	0.0204
Age, years	1.06	(1.04-1.08)	$1.00X10^{-8}$	1.05	(1.04-1.07)	$4.88X10^{-9}$
Steinbrocker stage, [1–4]	0.83	(0.70-0.97)	0.0178	0.79	(0.68-0.91)	0.0013
Smoking status [never smoker: 0, past smoker: 1, current smoker: 2]	1.58	(1.21-2.06)	0.0009	1.55	(1.20-2.00)	0.0008

RA: rheumatoid arthritis, CLD: chronic lung diseases, OR: Odds ratio, CI: confidence interval. *P*, OR, 95%CI, $P_{adiusted}$, $OR_{adiusted}$ were calculated by logistic regression analysis on RA patients.
