# Supplementary material for: Serum Anti-Aminoacyl-Transfer Ribonucleic Acid Synthetase Antibody Levels Are Involved in Rheumatoid Arthritis Complicated with Interstitial Lung Disease
Source: J Clin Med. 2024 Nov 10;13(22):6761. doi: 10.3390/jcm13226761 (PMC11594691; doi:10.3390/jcm13226761)
Supplement: Supplementary file 1 [file jcm-13-06761-s001.zip › Anti-ARS Ab#12Table-S4.pdf]

Supplementary Table S4. Multiple logistic regression analysis of anti-ARS Ab levels between RA patients and healthy controls.

Clinical manifestations	Unconditioned			Conditioned on the other factors		
	OR	95%CI	*P*	$OR_{adjusted}$	95%CI	$P_{adjusted}$
Anti-ARS Ab, Index	1.33	(1.16-1.52)	$3.24X10^{-5}$	1.20	(1.00-1.43)	0.0461
Age, years	1.19	(1.15-1.24)	$2.40X10^{-20}$	1.18	(1.14-1.23)	$4.01X10^{-18}$
Sex, male	7.23	(1.74-30.15)	0.0066	2.55	(0.42-15.48)	0.3104

RA: rheumatoid arthritis, OR: Odds ratio, CI: confidence interval. *P*, OR, 95%CI, $P_{adjusted}$, $OR_{adjusted}$ were calculated by logistic regression analysis in the comparison between RA patients and healthy controls.
